# Medial meniscus posterior root tear repair: An overview of surgical techniques and augmentation strategies

**DOI:** 10.1002/jeo2.70578

**Published:** 2026-01-11

**Authors:** Gabriele Cortina, Simone Perelli, Nicola Pizza, Vincenzo Condello, Vincenzo Madonna, Joan Carles Monllau

**Affiliations:** ^1^ Department of Orthopaedic, Joint Prosthetic, Arthroscopic Surgery and Sports Traumatology Humanitas Castelli Bergamo Italy; ^2^ Institut Català de Traumatologia i Medicina de l′Esport (ICATME)‐Hospital Universitari Dexeus, Universitat Autònoma de Barcelona Barcelona Spain; ^3^ Department of Surgery and Morphologic Science, Orthopaedic Surgery Service Universitat Pompeu Fabra, Hospital del Mar Barcelona Spain

**Keywords:** degenerative, extrusion, knee, meniscal extrusion, meniscus, osteoarthritis

## Abstract

**Purpose:**

Medial meniscus posterior root tears (MMPRTs) disrupt the hoop stress mechanism, leading to increased meniscal extrusion and early osteoarthritis. This systematic review aims to evaluate current surgical techniques for MMPRT repair, including their biomechanical principles, technical aspects and clinical outcomes, with special attention to strategies addressing meniscal extrusion.

**Methods:**

A systematic search was conducted across PubMed, Scopus and Cochrane Library using Preferred Reporting Items for Systematic Reviews and Meta‐Analyses (PRISMA) 2020 guidelines. Inclusion criteria encompassed studies describing surgical techniques for MMPRT repair published in the last 20 years. Techniques were categorized into pull‐out transtibial repair, suture anchor repair and all‐inside repair. Augmentation procedures were also evaluated. Quality assessment was performed using the Risk of Bias In Non‐randomised Studies of Interventions (ROBINS‐I) tool for comparative studies.

**Results:**

Forty‐two studies were included. Thirty studies reported on pull‐out transtibial techniques, eight on suture anchor repairs and four on all‐inside repair. Various fixation strategies, suture configurations and devices were described. Biomechanical evidence supports the use of suture anchors for improved cyclic loading performance. Augmentation techniques such as gracilis autografts or centralization sutures were described in select cases to reduce extrusion. Comparative studies suggest that while no single technique guarantees superior healing, suture anchor repair may offer biomechanical advantages over pull‐out repairs.

**Conclusion:**

Multiple surgical options exist for MMPRT repair, each with specific advantages and limitations. The choice of technique should consider biomechanical integrity, tissue quality and the presence of extrusion. Augmentation strategies may enhance outcomes in selected cases. Further high‐quality comparative studies are needed to define the optimal surgical approach.

**Level of Evidence:**

Level III, systematic review and meta‐analysis.

## Graphical abstract

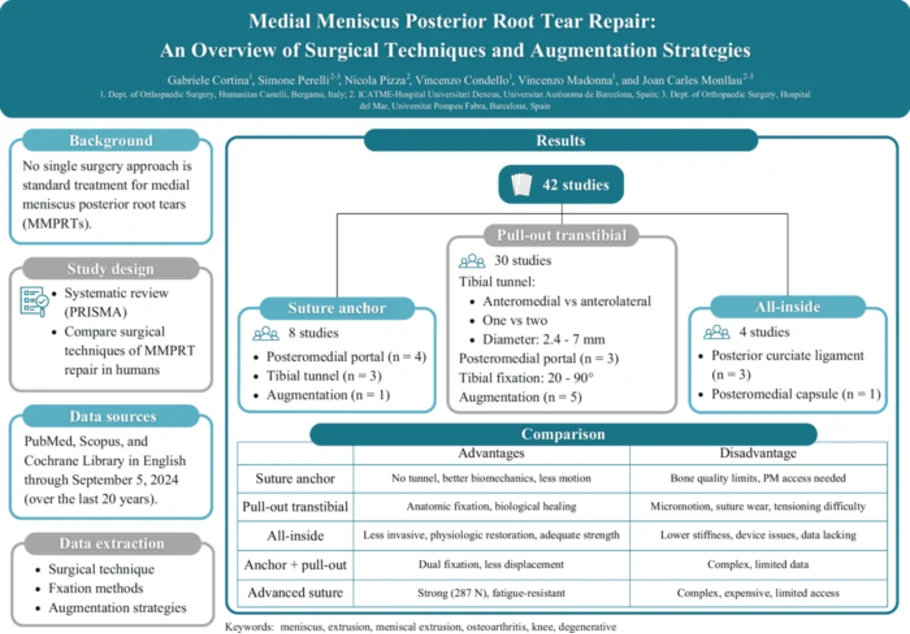

**Abbreviations**
D‐DLL: double‐double locking loop
F‐MMA: first modified Mason–Allen
IKDC: International Knee Documentation Committee
KOOS: Knee Injury and Osteoarthritis Outcome Score
MM: medial meniscus
MME: medial meniscus extrusion
MMPE: medial meniscus posterior extrusion
MMPR: medial meniscus posterior root
MMPRT: medial meniscus posterior root tear
OA: osteoarthritis
OWHTO: open‐wedge high tibial osteotomy
PA: posterior anchoring
PCL: posterior cruciate ligament
PM: posteromedial
PRISMA: Preferred Reporting Items for Systematic Reviews and Meta‐Analyses
S‐DLL: single‐double locking loop
TCS: two cinch stitches
TSS: two simple stitches
UHMWPE: ultrahigh‐molecular‐weight polyethylene
VAS: Visual Analogue Scale

## INTRODUCTION

**Figure 1:**
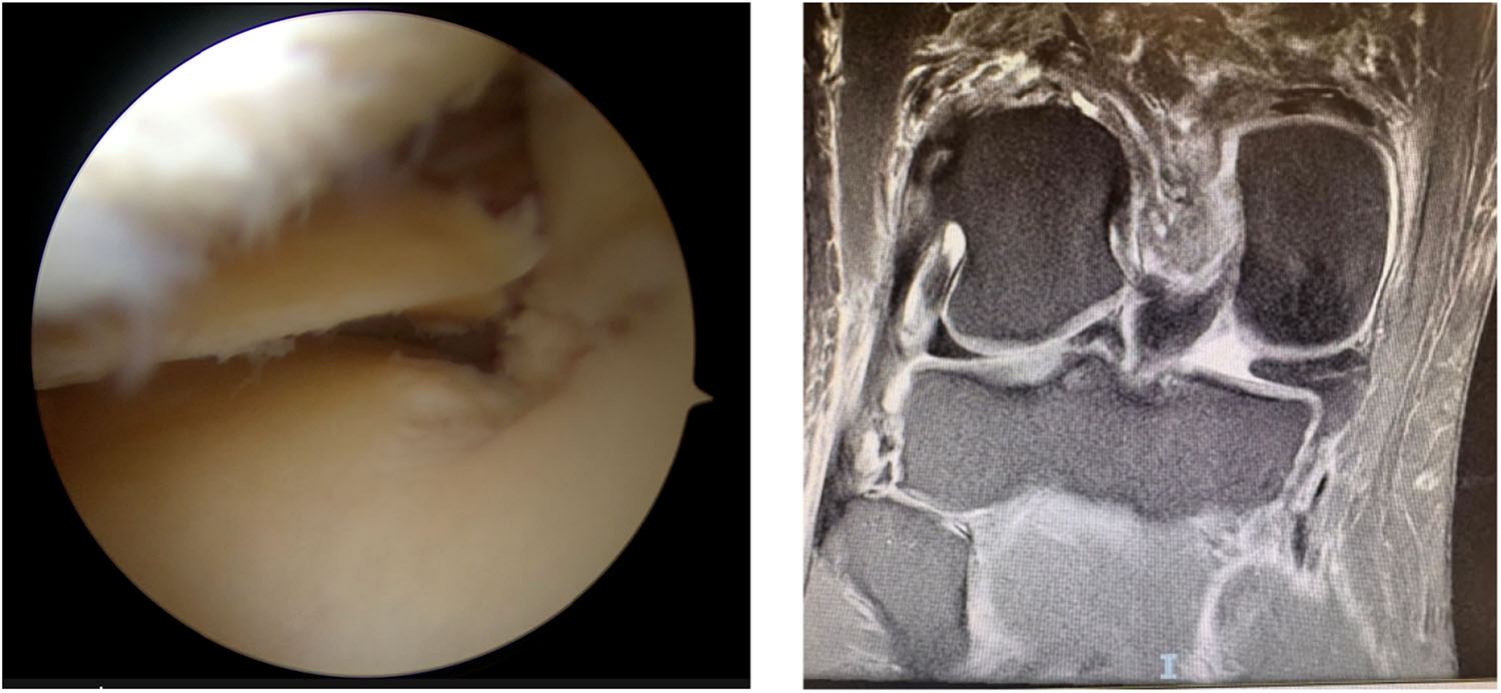
Arthroscopic view (left) showing a complete avulsion of the medial meniscus posterior root (MMPR). Coronal T2‐weighted magnetic resonance imaging (MRI) (right) demonstrates meniscal extrusion and disruption of the posterior root attachment, consistent with a MMPR tear.

Meniscal roots are the terminal extension of the circumferential collagen fibres that run at the periphery of the meniscal body and represent a fundamental connection of such structures to the tibial plateau [32]. Meniscal root tears are generally defined as lesions occurring within 9 mm from the meniscal insertion on the tibial plateau, according to the anatomical description proposed by LaPrade et al. [42] (Figure 1). The medial meniscus posterior root (MMPR) plays a crucial role in knee biomechanics, contributing to load distribution and joint stability, absorbing the axial load and resisting pull‐out forces [6]. The MMPR is fundamental, especially in the anteroposterior stability of the knee, compared to the lateral posterior root, which is more significant in the rotatory stability of the knee [59]. Pagnani et al. firstly described these lesions and recognised them as a substantial cause of knee pain and joint degeneration [66], with subsequent risks of early osteoarthritis (OA) [40]. Biomechanical studies have shown that MMPR tears result in a complete loss of hoop strain resistance (the so‐called ‘belt effect’) comparable to a total meniscectomy [2, 25].

Meniscal extrusion [33] is a condition frequently associated with injuries of the MMPR and represents an important prognostic factor of joint degeneration. Recent meta‐analyses have demonstrated that surgical centralization or extrusion reduction significantly improves joint biomechanics and clinical outcomes [7]. Biomechanical studies have demonstrated that injury to the MMPR not only causes the meniscus to lose its ability to resist hoop strain forces but also significantly increases meniscal extrusion, leading to altered tibiofemoral loading [23, 79].

For the aforementioned reasons, the MMPR repair has garnered increasing interest in the last few years [48]. The goal is to restore the biomechanical function of the meniscus and prevent further joint degeneration. Different strategies, including arthroscopic or open techniques [17], have been proposed, each with specific indications, advantages and limitations. However, despite the progress, a lively debate persists regarding the long‐term efficacy of these procedures and the choice of the optimal technique [1]. Even if the pull‐out transtibial repair and the suture anchor repair are the most widespread methods used for indirect or direct fixation of the root at the original footprint, some other strategies and different devices of fixation have been described in recent years [4, 45, 55]. The aim of the present systematic review is to meticulously analyse and summarise the existing literature on surgical techniques for repairing MMPR tears. A rigorous critical analysis of the most recent studies aims to provide an in‐depth overview of the available devices and materials to restore this fundamental knee stabilizer with the goal of pointing out the advantages and disadvantages of the different techniques from a biomechanical and surgical point of view. It was hypothesized that, given the diversity of available approaches, no single repair technique can currently be considered superior; instead, each surgical strategy presents specific biomechanical and technical advantages that must be interpreted in relation to meniscal extrusion control [67] and tissue quality.

## MATERIALS AND METHODS

A literature review was performed using a search strategy designed to collect articles regarding the surgical repair of MMPR tears. The inclusion criteria adopted were as follows: (1) studies that explain a surgical technique for MMPR repair meticulously; (2) articles published within the last 20 years; (3) studies published in English and (4) studies only involving the human species (including cadaveric studies). Review articles were excluded from the search. The Preferred Reporting Items for Systematic Reviews and Meta‐Analyses (PRISMA) 2020 flow diagram was used to conduct the systematic review. Two reviewers (G. C. and N. P.) conducted an electronic systematic search on Scopus, PubMed and Cochrane Library to find eligible studies. The investigation was conducted on 5 September 2024. The following search string was adopted: (((meniscal posterior medial Root injury) OR (meniscal posterior medial Root lesion) OR (meniscal posterior medial root)) OR (medial posterior root lesion)) AND ((operative technique) OR (surgical technique) OR (repair) OR (suture) OR (management) OR (technical note))). The search strategy was collaboratively developed by the authors to enhance the specificity for studies explicitly describing surgical repair techniques of MMPR tears. Broader terms such as ‘posterior horn avulsion′ were initially considered but ultimately excluded to prevent retrieving unrelated meniscal injury patterns.

Each study was evaluated according to its inclusion criteria. After deleting the duplicates, the relevant full‐text articles from the electronic search were obtained and assessed. The bibliography of each study was manually searched to find any potentially pertinent papers. Finally, the two reviewers (G. C. and N. P.) examined the remaining manuscripts to select the included studies. Two authors (S. P. and G. C.) extracted these data from each paper: authors, type of study with the level of evidence, number of patients, tibial tunnel location, number of tibial tunnels, the use of posteromedial (PM) portal, tibial guide, implant for tibial fixation, type of knot with material, type of suture passer, any augmentation and the angle of fixation.

### Quality assessment of included studies

The ROBINS‐I tool (‘Risk of Bias In Non‐randomised Studies of Interventions’) was used to rate the quality of comparative studies included in the current systematic review. Two authors (G. C. and N. P.) independently evaluated the quality of each study.

## RESULTS

**Figure 2:**
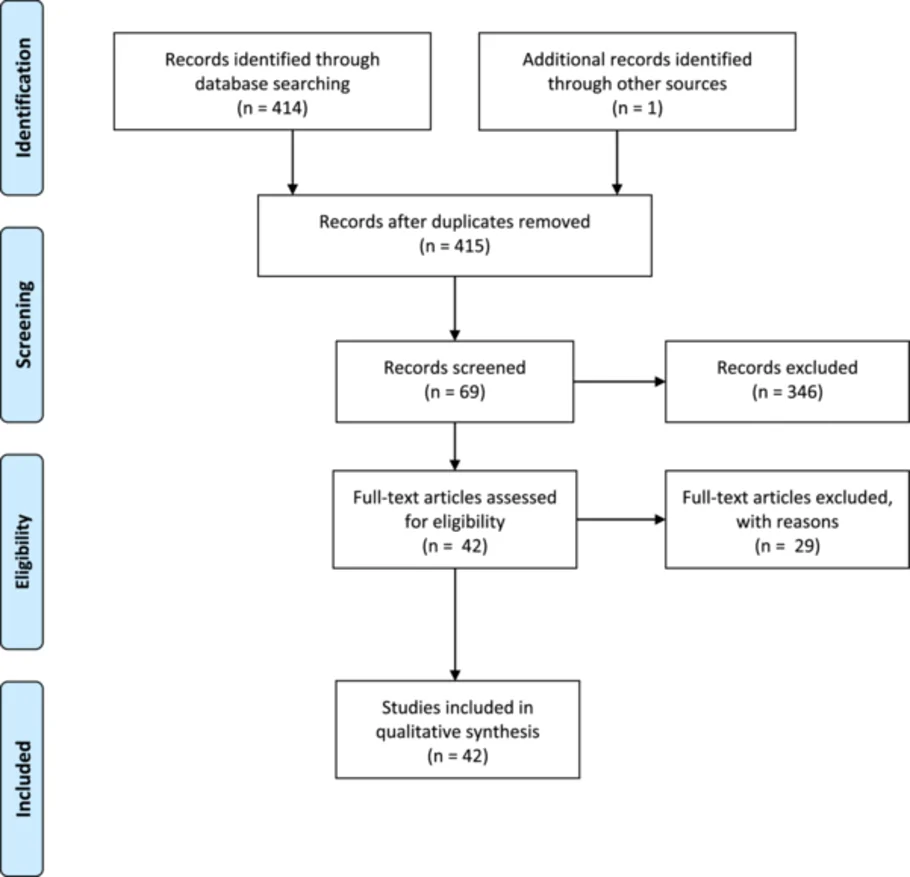
PRISMA flowchart. PRISMA, Preferred Reporting Items for Systematic Reviews and Meta‐Analyses.

After the exclusion process detailed in Figure 2, 42 studies were included in the current systematic review.

### Suture anchor repair

Among the studies included (Table 1), eight described a suture anchor technique for MMPR repair [5, 12, 38, 51, 56, 57, 61, 74]. Four studies described using a PM portal to introduce the suture anchor in the tibial footprint [12, 51, 57, 61]. Meanwhile, three studies performed a tibial tunnel to fix the anchor retrogradely. In the study of Meng et al., after the insertion of a 2.0‐mm Kirschner wire from the anteromedial tibial cortex, a 4.5‐mm drill bit is used to create a broad bone tunnel, stopping approximately 1–1.5 cm away from the posteromedial tibial articular cartilage surface with the guidance of the equivalent‐length Kirschner wire. This creates a tibial tunnel with an upper part that is narrower and a lower part that is broader; the junction between them is used to fix the suture anchor [56]. Srimongkolpitak et al. described in a similar fashion the insertion of a suture anchor retrogradely from the anteromedial tibial cortex after the creation of a 2.9 mm tibial tunnel [74]. Balke et al. used a suture anchor typically used in shoulder surgery to create a 1.8 mm tibial tunnel in accordance with the guidelines of the manufacturing company [5].

**Table 1:** Baseline characteristics of included studies, suture anchor cohort.

Study	Design	Use of PM portal	Suture anchor	Type of knot (material)	Suture passer	Augmentation
Choi et al. [12]	Technical note	Yes	Metal suture anchor, loaded with two fibre wires (Arthrex)	Mattress suture configuration + SMC knots	Curved suture hook (Linvatec)	
Kuptniratsaikul et al. [38]	Technical note	No	JuggerKnot 1.4 anchor (Biomet Sports Medicine)	NR	FIRSTPASS suture passer (Smith & Nephew)	
Lee et al. [49]	Technical note	Yes	2.3‐mm ICONIX soft suture anchor (Stryker Endoscopy) double‐loaded with no. 2 force fibre (Teleflex Medical OEM)	SMC knot	Curved suture hook (Linvatec)	
Meng et al. [56]	Technical note	No	3.0‐mm anchor (Johnson & Johnson) inserted in a reverse direction for the anteromedial tibial cortex	Standard surgical knot	NR	
Mesquita Queiros et al. [57]	Technical note	Yes	1.6 knotless FiberTak soft anchors (Arthrex)	Mattress suture configuration	Knee Scorpion suture passer (Arthrex)	
Nakamura et al. [61]	Technical note	Yes	An anchor for the lateral row (FiberTak; Arthrex) with two sutures and a Q‐FIX anchor for the medial row	NR	Knee Scorpion suture passer (Arthrex)	Centralization with JuggerKnot Soft Anchor (Zimmer Biomet Japan)
Srimongkolpitak et al. [74]	Technical note	No	Triple‐loaded soft anchor (Y‐Knot PRO RC, 2.8 mm, ConMed) inserted in a reverse direction for the anteromedial tibial cortex	Mason–Allen suture configuration + two simple stitches	FirstPass Mini Suture Passer (Smith & Nephew)	
Balke et al. [5]	Technical note	No	Y‐Knot Flex 1.8‐mm disposable drill bit (Conmed) from tibial tunnel in retrograde direction	Simple stitch + lasso‐loop stitch	NR	

Abbreviations: NR, not reported; PM, posteromedial portal; SMC, Samsung Medical Center.

There is a high degree of variability regarding the type of knot used for fixing the MMPR. Three studies described the use of Modified Mason–Allen configuration [56, 72, 74]; two studies used a mattress configuration [12, 57]; one study used the Samsung Medical Center (SMC) knot [51]; one study described the use of simple stitch plus lasso‐loop technique [5]. Only one study described the centralization technique as an augmentation of the MMPR repair [61]. Although augmentation with suture anchors has been described, evidence supporting its clinical or biomechanical superiority remains extremely limited. The current paucity of comparative data does not allow any firm recommendation regarding augmentation when using anchor‐based repair techniques.

### Pull‐out transtibial repair

Among the studies included (Table 2), thirty described a pull‐out transtibial technique for MMPR repair [11, 19, 21, 22, 24, 26, 29, 35, 36, 39, 43, 47, 49, 50, 52, 53, 58, 60, 62, 64, 68, 69, 71, 73, 78, 80, 82, 83]. Nineteen were technical notes [11, 24, 28, 29, 36, 47, 49, 50, 52, 53, 64, 68, 73, 78, 80], eight retrospective studies [21, 22, 26, 27, 35, 39, 64, 83], two controlled laboratory studies [43, 60] and one case series study [58].

**Table 2:** Baseline characteristics of included studies, pull‐out cohort.

Study	Design	Patients	Tibial tunnel location (no. of tibial tunnels)	Tibial tunnel diameter (mm)	Use of PM portal	Tibial guide	Tibial fixation	Type of knot (material)	Suture passer	Augmentation	Pull‐out + anchor	Angle of fixation
Lee et al. [51]	Technical note		Medial (1)	NR		NR	Hewson button (Ethicon)	Mason–Allen stitch (no. 1 PDS, [Ethicon])	Crescent‐shaped suture hook (Linvatec)			30°–45° knee flexion
Furumatsu et al. [23]	Retrospective cohort study	38	Medial (1)	4.0		Unicorn Meniscal Root (UMR) (Arthrex Inc)	5.0 × 20‐mm interference screw	One simple stitch (no. 2 FiberWire [Arthrex Inc])	Knee Scorpion suture passer (Arthrex)			20° knee flexion + 30 N of tension
						MMPRT guide (Smith & Nephew)						
Kwon et al. [40]	Retrospective cohort study	51	Medial and lateral (1)	NR		ACL tibial guide (Arthrex Inc)	NR	NR	NR			NR
Hiranaka et al. [27]	Retrospective cohort study	68	NR	NR		NR	Double‐spike plate and interference screw	Mason–Allen stich (no. 2 Ultrabraid [(Smith & Nephew)] + FasT‐Fix 360 meniscal repair system [(Smith & Nephew)])	Knee Scorpion suture passer (Arthrex)			45° knee flexion
								Two simple stitches (no. 2 Ultrabraid [Smith & Nephew])				20° knee flexion
Cho [12]	Technical note		Medial (1)	6.0	Yes	ACL tibial guide (Linvatec)	6.5 mm cancellous bone screw and smooth washer	Two simple stitches (no. 0 PDS, [Ethicon])	Crescent‐shaped suture hook (Linvatec)			NR
LaPrade et al. [43]	Controlled laboratory study		Medial (1 vs. 2)	2.4		MMPRT guide (Smith & Nephew)	Endobutton (Smith & Nephew)	Two simple stitches (no. 2 Ultrabraid [Smith & Nephew])	NR			NR
Mameri et al. [54]	Technical note		Medial (2)	2.7		MMPRT guide (Smith & Nephew)	FOOTPRINT Suture Anchor (Smith & Nephew)	Two simple stitches (high‐strength suture tape)	FIRSTPASS MINI (Smith & Nephew)			90° knee flexion
Kintaka et al. [36]	Retrospective cohort study	35	Medial (1)	NR		MMPRT guide (Smith & Nephew)	Double‐spike plate and cancellous screw (Meira)	Mason–Allen stitch (no. 2 Ultrabraid [Smith & Nephew] vertically through the meniscal tissue + The FasT‐Fix 360 meniscal repair system [Smith & Nephew])	Knee Scorpion suture passer (Arthrex)			45° knee flexion + 20 N tension using a spring tensioner
							Bioabsorbable interference screw (Biosure RG, Smith & Nephew) and a cancellous screw (Meira)	Two no. 2 Ultrabraid sutures vertically (Smith & Nephew)				20° knee flexion + 30 N of tension using a spring tensioner
Nakama et al. [61]	Controlled laboratory study		Medial (1)	2.4		MMPRT guide (Smith & Nephew)	Endobutton (Smith & Nephew)	Two simple stitches (UHMWPE suture tape [Ultratape; Smith & Nephew])	FIRSTPASS MINI (Smith & Nephew)			NR
								Two simple stitches (no. 2 Ultrabraid [Smith & Nephew])				
								Two simple stitches (no. 0 ETHIBOND; Ethicon)				
van der List et al. [53]	Technical note		Medial (1)	2.4		Meniscal root guide (Arthrex)	SutureLoc System (Arthrex)	One horizontal mattress (FiberWire [Arthrex Inc])	Knee Scorpion suture passer (Arthrex)			NR
Okazaki et al. [65]	Technical note		Medial (1)	4.0		MMPRT guide (Smith & Nephew)	Bioabsorbable interference screw	Two simple stitches (no. 2 Ultrabraid and/or Ultratape (Smith & Nephew) + The FasT‐Fix 360 meniscal repair system (Smith & Nephew) in the posteromedial aspect of MM	Knee Scorpion suture passer (Arthrex)			20° knee flexion + 30 N of tension using a spring tensioner
Kodama et al. [37]	Technical note		Medial (1)	4.0		MMPRT guide (Smith & Nephew)	Double‐spike plate and screw (Meira)	One horizontal mattress (FasT‐Fix 360 meniscal repair system [Smith & Nephew])	NR			40° knee flexion
Mochizuki et al. [59]	Case series	26	Medial (1)	4.5		MMPRT guide (Smith & Nephew)	Endobutton (Smith & Nephew)	NR	Knee Scorpion suture passer (Arthrex)	Centralization with two suture anchors (1.7 mm SUTUREFIX ULTRA, Smith & Nephew)		NR
Lee et al. [50]	Technical note		Medial (1)	6.0		ACL tibial guide (Linvatec)	5.5 interference screw	NR	Crescent‐shaped suture hook (Linvatec)	Gracilis Autograft		45° knee flexion
Holmes et al. [29]	Technical note		Medial (1)	NR		Meniscal root guide (Arthrex)	4.75‐mm PEEK (polyether ether ketone) knotless suture anchor (SwiveLock; Arthrex)	Two horizontal mattress (no. 2 suture tape (SutureTape; Arthrex) + collagen‐coated braided nylon suture tape (InternalBrace; Arthrex))	Knee Scorpion suture passer (Arthrex)	Gracilis Autograft		30° knee flexion
Lavender et al. [48]	Technical note		Medial (1)	6.0		ACL guide (Arthrex)	3.5‐mm suture button (Arthrex)	Modified Mason–Allen stitch (Orthocord suture [DePuy Mitek])	Suture‐passing hook (ConMed)			NR
Ishikawa et al. [30]	Technical note		Medial (1)	6.0	Yes	ACL tibial guide (3 M)	Pull‐out button (AI‐Medic)	Two simple stitches (no. 2 Ultrabraid, Smith & Nephew)	Meniscal Viper (Arthrex)	Gracilis Autograft	1.8‐mm all‐suture anchor (Q‐FIX MINI, Smith & Nephew)	NR
Hiranaka et al. [28]	Retrospective cohort study	36	Medial (1)	4.0		MMPRT guide (Smith & Nephew)	Interference screw	Two vertical sutures (Ultrabraid [Smith & Nephew] and FiberWire [Arthrex])	Knee Scorpion suture passer (Arthrex)		30° knee flexion with 30 N of tension with spring tensioner	
								Two vertical stitches (UHMWPE suture tapes)				
Tahami et al. [79]	Technical note		Medial (1)	4.3		NR	Screw	‘Loop‐Post Construct’ (no. 0 FiberWire [Arthrex])	Suture hook EZpass 70 degrees (Zimmer‐Biomet)			30° knee flexion
Woodmass et al. [81]	Technical note		Medial (1)	6.0		Point‐to‐Point Marking Hook (Arthrex)	4.75‐mm PEEK (polyether ether ketone) knotless suture anchor (SwiveLock; Arthrex)	Two cinch knots + one simple stitch (FiberWire [Arthrex])	Knee Scorpion suture passer (Arthrex)			30° knee flexion
Goes et al. [25]	Technical note		Lateral (1)	6.0		Multiuse (MU) guide (Arthrex)	ABS suture button (Arthrex)	Two cinch knots (FiberWire [Arthrex])	Knee Scorpion suture passer (Arthrex)			NR
Samy et al. [74]	Technical note		Lateral (1)	7.0		Tibial ACL drill guide (Arthrex)	Washer	Two cinch knots (FiberWire [Arthrex])	A suture‐passing lasso device (Quick pass suture Lasso, Arthrex)			15° knee flexion
Okazaki et al. [63]	Retrospective cohort study	37	Medial (1)	4.0		MMPRT guide (Smith & Nephew)	Interference screw	Two vertical stitches of UHMWPE suture tape (Ultrabraid [Smith & Nephew] and FiberWire [Arthrex])	Knee Scorpion suture passer (Arthrex)			30° knee flexion with 30 N of tension with spring tensioner
								Two vertical stitches of polyester suture (Macaroni, Stryker Japan KK)				
Revelt et al. [70]	Technical note		Medial (1)	NR		Meniscal root repair guide (Arthrex)	Suture button (Arthrex)	Two vertical stitches (FiberWire [Arthrex])	Knee Scorpion suture passer (Arthrex)			NR
Drynan et al. [20]	Technical note		Lateral (1)	2.4		ACL ‘tip’ tibial guide (Smith & Nephew)	EndoButton (Smith & Nephew)	One ‘Luggage‐Tag’ + Horizontal Mattress Sutures (no. 2 Ultrabraid [Smith & Nephew])	45° ConMed Spectrum suture passer (ConMed)			30° knee flexion
Perelli et al. [69]	Technical note		Lateral (1)	4.5		MMPRT guide (Smith & Nephew)	Biceps Button (Arthrex)	Two cinch knots (no. 2 Ultrabraid suture (no. 2 Ultrabraid suture ([Smith & Nephew])	FIRSTPASS MINI (Smith & Nephew)			90° knee flexion
Wu [83]	Technical note		Medial (1)	2.4		ACL ‘tip’ tibial guide (Smith & Nephew)	4.75 mm PEEK (polyether ether ketone) SwiveLock anchor (Arthrex)	Two cinch knots (FiberWire [Arthrex])	Knee Scorpion suture passer (Arthrex)	Centralization with 3 mm Knotless BioComposite SutureTak anchor (Arthrex)		NR
Furumatsu et al. [22]	Retrospective cohort study	83	Medial (1)	4.0		MMPRT guide (Smith & Nephew)	Double‐spike plate or interference screw	Modified Mason–Allen suture (Ultrabraid [Smith & Nephew]) + FasT‐Fix all‐inside meniscal repair device (Ultrabraid [Smith & Nephew])	Knee Scorpion suture passer (Arthrex)			20°–45° knee flexion with 20 N of tension
								Two simple stitches (no. 2 polyethylene sutures Ultrabraid [Smith & Nephew] or FiberWire [Arthrex])				
								Two simple stitches (no. 2 polyethylene sutures Ultrabraid [Smith & Nephew] or FiberWire [Arthrex]) + FasT‐Fix all‐inside meniscal repair device (Ultrabraid [Smith & Nephew])				
Xue et al. [84]	Retrospective cohort study	31	Medial (1)	4.0		MMPRT guide (Smith & Nephew)	Bioabsorbable screw	Two‐cinch stitches (no. 2 Ultrabraid [Smith & Nephew])	Knee Scorpion suture passer (Arthrex)		JuggerStitch™ meniscal repair device (Zimmer Biomet)	30° knee flexion with 10 N of tension
								Two‐cinch stitches (no. 2 Ultrabraid [Smith & Nephew])				
Rocha de Faria et al. [72]	Technical note		Medial (1)	6.0	Yes	Multiuse guide (Arthrex)	EndoButton (Smith & Nephew)	Three Mickey's ear stitches (no. 2‐0 FiberWire [Arthrex])	The Meniscus 4 A‐II device			NR

Abbreviations: ACL, anterior cruciate ligament; MMPRT, medial meniscus posterior root tear; NR, not reported; PEEK, polyether ether ketone; UHMWPE, ultrahigh‐molecular‐weight polyethylene.

Most included studies performed the tibial tunnel from the anteromedial tibial cortex for the pull‐out transtibial repair. Four studies reported performing the tibial tunnel from the anterolateral cortex of the tibia [19, 24, 68, 73]. Goes et al., particularly, highlight the advantages of creating the tibial tunnel from the anterolateral tibial cortex in cases of concomitant open‐wedge high tibial osteotomy (OWHTO). This approach reduces the risk of the tibial tunnel converging with the most proximal screws of the osteotomy plate. Furthermore, this positioning establishes a more natural angle for the suture threads, thereby reducing the ‘killer angle’ [24]. One study compared the creation of the tibial tunnel through the anteromedial versus the anterolateral tibial cortex. Notably, Kwon et al. demonstrated that tunnelling from the anterolateral side results in more accurate restoration of the native meniscal root footprint compared to the anteromedial approach. On average, the anteromedial technique led to a medial deviation of the tunnel relative to the anatomic footprint [39].

Most studies described a single tibial tunnel, while one technical note describes the use of two tibial tunnels from the anteromedial cortex of the tibia for MMPR Pull‐out Transtibial repair [53]. Moreover, only one study analysed the biomechanical difference between one and two tibial tunnels in a cadaveric setting. It showed no statistical differences between the one and two tibial tunnels for displacement or ultimate failure load [43].

Regarding the diameter of the tibial tunnel used for MMPR pull‐out transtibial repair, a wide variability was observed in the included studies, with sizes ranging from 2.4 mm [19, 43, 52, 60, 82] to 7.0 mm [73]. The reasons for this variability in tunnel calibre are derived from the use of an augmentation, such as gracilis autograft [29, 49] or in order to increase the accommodation of meniscal tissue within the tunnel itself to promote healing [73]. However, the correlation between tunnel size with clinical outcomes and biomechanical performance could not be identified, likely due to methodological heterogeneity among studies. Furthermore, a recent systematic review suggests that suture configuration and precise tunnel placement, rather than size per se, are the main determinants of biomechanical success and clinical improvement [8].

Three studies described the use of a PM portal [11, 29, 71]. Cho used the PM to prepare the tibial footprint and create the tibial tunnel [11]. Ishikawa et al. described using a PM portal to augment the MMPR pull‐out repair with a suture anchor [29]. Rocha de Faria and colleagues introduced a meniscal suture device [71] using the PM portal.

Regarding the tibial fixation, the current systematic review showed no consensus regarding the degrees of knee flexion. In fact, the range of tibial fixation ranges from 20° [27] to 90° of knee flexion [68]. Moreover, there is a wide choice of devices used for fixation that can be categorised into interference screws [21, 22, 26, 27, 35, 62, 64, 83], screws [11, 35, 36, 78], nontensionable buttons [19, 24, 29, 43, 47, 50, 60, 69, 71, 73], tensionable buttons [52, 53, 68] and anchors [28, 80, 82].

### Augmentation

In the current systematic review, six studies described MMPR repair techniques using augmentation techniques. Specifically, three studies described the use of an autologous gracilis tendon in cases of retracted and/or chronic MMPR tears with very low mobility [28, 29, 49]. Three studies [59, 83, 84] further described a repair technique that aims to reduce meniscal extrusion, which is almost always associated with MMPR. Mochizuki et al. augmented the MMPR repair with a centralization technique using two suture anchors at the body of the medial meniscus [58]. Ishikawa et al. simultaneously described the use of gracilis autograft augmentation for chronic MMPR tears and the placement of a suture anchor at the posteromedial edge of the tibial plateau [29]. Xue et al. described how to address meniscal extrusion by adding a suture anchor to the posteromedial edge of the tibial plateau in a transtibial pull‐out repair [83].

### All‐inside repair

Among the included studies (Table 3), four studies describe the repair of the posterior root of the medial meniscus using the all‐inside technique [18, 31, 72, 76]. Specifically, one biomechanical study [73], two retrospective studies [18, 31] and one cohort study [77] were included. A total of 79 patients were included. Three studies reported that, using an all‐inside repair system, root repair is performed by fixation to the posterior cruciate ligament (PCL) [31, 72, 76]. Meanwhile, one study described the fixation of MMPR to the posteromedial capsule [18]. In the included studies, no major complications such as PCL injury or fixation failure were reported.

**Table 3:** Baseline characteristics of included studies, all‐inside cohort.

Study	Design	No of patients	Use of PM portal	PCL fixation	Device
Su et al. [76]	Cohort study		No	Yes	Two FAST‐FIX 360 meniscal repairing systems (Smith & Nephew Endoscopy)
Saltzman et al. [72]	Controlled laboratory study		No	Yes	Vertical and horizontal mattress (No. 2 nonabsorbable suture [Arthrex])
Jiang et al. [31]	Retrospective cohort study	20	No	Yes	Two FAST‐FIX 360 meniscal repairing systems (Smith & Nephew Endoscopy)
Dragoo et al. [18]	Retrospective cohort study	30	No	No	Three FAST‐FIX 360 meniscal repairing system (Smith & Nephew Endoscopy)

Abbreviations: PCL, posterior cruciate ligament; PM, posteromedial portal.

### Comparative studies

Furumatsu et al., in a retrospective study, compared the position of the tibial tunnel created between two meniscal root repair guides. The main difference is the catching point, one at the tip of the guide, the other at the neck of the hook. The mean distances between the tunnel centre and the anatomic centre were 4.06 and 3.99 mm between the two guides used (*p* = 0.455), with no significant differences between the groups [22].

Hiranaka et al., in a retrospective study, compared two simple ultrabraid stitches (TSS) versus a single ultrabraid suture plus a modified Mason–Allen suture with an ultrabraid all‐inside meniscal repair system (F‐MMA technique). No significant difference was seen in the clinical outcome scores and the meniscal healing status between the two groups at second‐look arthroscopy [26].

Hiranaka et al., in a retrospective study, compared a Mason–Allen stitch with an ultrahigh molecular‐weight polyethylene (UHMWPE) + an all‐inside meniscal repair system with two vertical stitches with UHMWPE suture tapes. No significant differences in second‐look arthroscopy were recorded, but an arthroscopic meniscal healing score significantly differed between sutures (mean 6.7 points) and suture tape (mean 7.4 points; *p* = 0.044) [27].

Kintaka et al., in a retrospective study, using the same suture and configuration setting as Hiranaka et al. [26], showed that the TSS technique, without grasping the posterior capsule, was better at preventing the progression of postoperative medial meniscus posterior extrusion (MMPE), especially in knee extension, than the F‐MMA technique [35].

Okazaki et al., in a retrospective study, compared the clinical efficacy of suture materials using polyester hollow sutures versus UHMWPE suture tapes. They showed significantly higher meniscal healing scores and decreased Visual Analogue Scale (VAS) pain scores in the UHMWPE group [62].

Xue et al., in a retrospective study, analysed the role of additional posterior anchoring (PA) during pull‐out transtibial repair in reducing the severity of MMPE compared to pull‐out transtibial repair alone. A two‐cinch stitch configuration with or without a suture anchor in the posteromedial corner of the medial meniscus was used. Both techniques improved MMPE at knee flexion at the last follow‐up, with a PA showing significantly superior results (*p* < 0.05) [83].

Furumatsu et al., in a retrospective study, compared three surgical techniques: an F‐MMA technique, two simple stitches (TSS) and TSS concomitant with posteromedial pull‐out transtibial repair using an all‐inside meniscal repair device. They showed that at second‐look arthroscopies, there were no significant differences between the three techniques in postoperative clinical outcomes and meniscal healing scores [21].

Kwon et al., in a retrospective study, compared the differences in outcome and tunnel placement between medial and lateral placement. They showed no statistically significant difference in changes in the International Knee Documentation Committee (IKDC) and Lysholm score between the two cohorts of tunnel placement; however, the study showed a slight but statistically significant medialization of the MMPR after the operation in the cohort of patients with a medial tibial tunnel [39].

Although these retrospective comparisons provide valuable insights into different surgical approaches, the marked heterogeneity in study design, patient selection and sample size precludes drawing firm comparative conclusions. Therefore, the reported differences in outcomes should be interpreted with caution.

### Quality assessment

**Figure 3:**
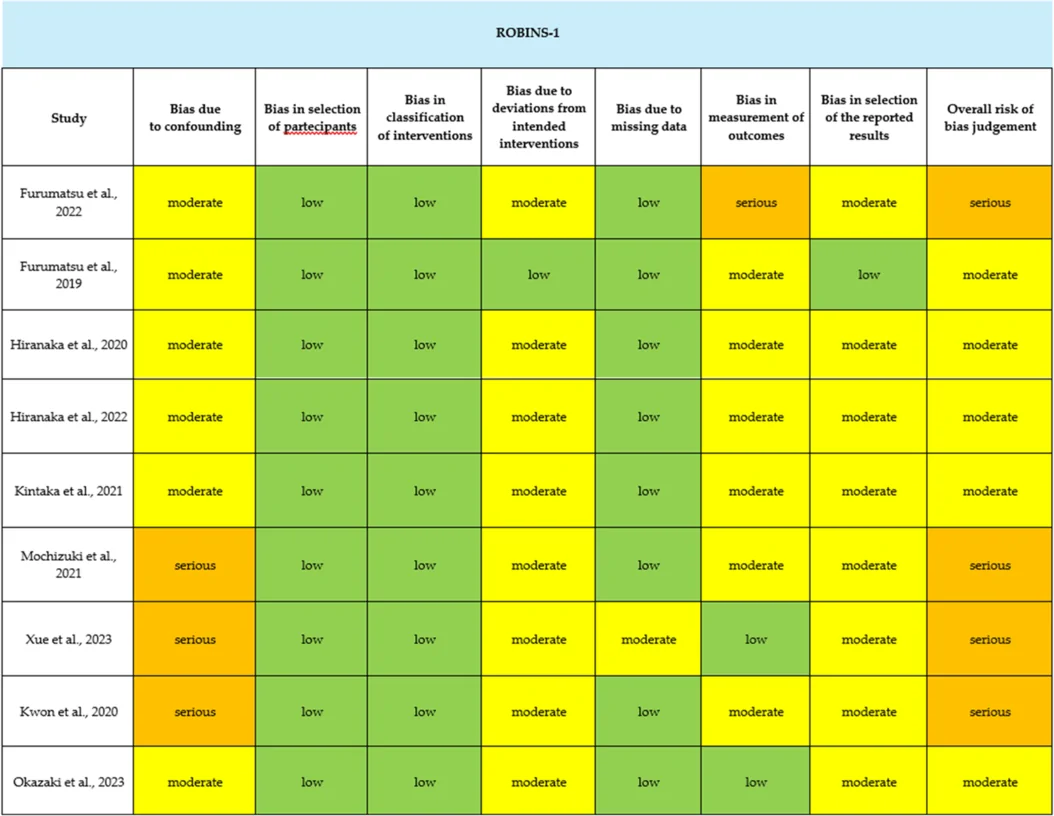
Quality assessment of the comparative studies included.

The risk of bias of the comparative included studies was evaluated using the ROBINS‐I tool. Among the included studies, none were classified as having a low risk of bias; five studies presented a moderate risk of bias [22, 26, 27, 35, 62], and four studies were rated as having a serious risk of bias [21, 39, 58, 83]. No studies were judged as having a critical risk. The domains most frequently associated with a moderate or serious risk of bias were confounding and measurement of outcomes. Detailed results for the quality assessment of each included study are reported in Figure 3.

## DISCUSSION

The present systematic review examines the surgical techniques reported in the literature for repairing MMPR tears. Several techniques have been described and can be classified into three main categories: anchor repair, pull‐out transtibial repair and all‐inside repair. The medial meniscus root is an essential structure because tears at or close to the root attachment have been reported to significantly alter tibiofemoral contact mechanics, leading to accelerated progression of medial compartment OA [46]. Surgical outcome of MMPR repair provides functional and clinical benefits which could persist over the mid‐ to long‐term follow‐up [15, 16]. Nowadays, surgical repair cannot completely prevent degenerative changes in the knee joint, but less severe OA compared to that in nonoperative treatment or meniscectomy has been shown [10].

Anyway, the outcomes following MMPR repair are multifactorial; in fact, many features related to the patients and their knees should be considered, such as age at the time of surgery [13], acute o chronic lesion, obesity [84], the posterior tibial slope, the coronal alignment and concomitant chondral lesions in the medial compartment (especially outerbridge classification > 3) [14, 63]. Intraoperatively, the degree of degeneration of the posterior horn of the medial meniscus, the tear gap size and the reducibility should also be considered before MMPR repair [30].

Several studies have analysed which technique is preferable, especially between anchor‐ and pullout‐based. Kim et al., in a prospective therapeutic comparative study, evaluated the clinical and radiologic results of arthroscopic suture anchor repair versus pull‐out transtibial repair of MMPR in 45 patients. After a mean follow‐up of 26 months, both groups showed significant improvements in function (*p* < 0.05). They did not show significant differences in Kellgren–Lawrence grade (*p* < 0.05) compared with preoperatively and with a similar reduction of the meniscal extrusion. However, on postoperative magnetic resonance imaging (MRI) evaluation, significantly more cases of incomplete healing were observed in the pull‐out transtibial group [34].

Meniscal extrusion, often accompanying MMPR tears, is a crucial prognostic factor due to its impact on tibiofemoral load distribution and subsequent cartilage degeneration. While root repair may partially reduce extrusion, complete correction is rarely achieved. Persistent extrusion has been linked to worse clinical outcomes and faster OA progression. In the reviewed literature, several techniques have been proposed to address this issue concurrently with root repair [41]. Centralization procedures using additional suture anchors to tether the meniscus to the tibial plateau have shown promising results in reducing extrusion and improving meniscal positioning [8]. Furthermore, recent biomechanical studies support the hypothesis that PA combined with pull‐out repair significantly improves extrusion control compared to pull‐out repair alone [4]. Given its strong association with joint preservation and functional outcomes, reduction of meniscal extrusion should be regarded as a primary surgical goal during repair, particularly in patients with preoperative severe extrusion or varus alignment.

Also, Omae et al. recently compared the outcomes of pull‐out transtibial repair (*P* group) and suture anchor repair (SA group) for MMPR tears in patients undergoing OWHTO. At the second‐look arthroscopy, the SA group showed a significantly higher rate of complete healing (64.9%) than the *P* group (21.6%, *p* < 0.001). After a 2‐year follow‐up, the SA group had a significantly higher Lysholm score (89.6 ± 10.7) than the *P* group (80.9 ± 17.4, *p* = 0.011) [65]. From a biomechanical perspective, Feucht et al., in 24 fresh‐frozen porcine tibiae, compared the pull‐out transtibial repair (TP) and suture anchor repair (SA). They highlighted the superiority of the SA technique under cyclic loading conditions and load‐to‐failure testing compared with the TP technique. At the same time, both techniques showed inferior biomechanical properties compared with the native MMPR [20]. This study has highlighted how, beyond the biological factors that may intervene within the root repair processes, the biomechanical properties of the repair technique may also play an important role. The pull‐out transtibial repair technique may have several disadvantages. Indeed, the sutures on the tibial cortex create a long meniscus‐suture construct, which can provide micromotion of the repaired root in the early postoperative period, undermining the healing processes [70].

Furthermore, within the bone tunnel, abrasion of the suture material might develop before meniscal healing. In addition, adequate tensioning of the sutures is difficult because of the long distance of the sutures in the tibial tunnel [20]. In theory, the suture anchor repair eliminates these disadvantages and allows for direct refixation of the MMPR at its tibial insertion site.

Although the available clinical studies are heterogeneous and mostly retrospective, certain trends can be observed. Suture anchor repairs tend to demonstrate superior or comparable functional outcomes (Lysholm, IKDC) and higher healing rates compared to pull‐out repairs. Pull‐out repairs with additional PA or centralisation sutures also appear to reduce meniscal extrusion more effectively than standard pull‐out techniques. Therefore, despite promising findings, the low level of evidence and methodological heterogeneity prevent definitive recommendations.

The all‐inside technique represents a less invasive alternative to traditional transtibial pull‐out or anchor‐based repairs. Four studies [3, 31, 72, 76] described all‐inside MMPR repair, with most employing fixation to the PCL or, in one case, to the posteromedial capsule [18]. This method avoids tibial tunnels, potentially reducing operative time and tunnel‐associated complications. While early results appear favourable in terms of pain relief and functional recovery, biomechanical concerns remain, particularly regarding fixation strength and anatomical restoration of hoop stress. Moreover, PCL‐based fixation may not replicate native root biomechanics and could theoretically lead to altered load transfer. Given these limitations, all‐inside repairs may be considered in select patient populations—such as elderly patients or those with lower functional demands—but require further investigation through high‐quality comparative studies.

The biomechanical performance of medial meniscus posterior root tear (MMPRT) repairs largely depends on both the suture configuration and the mechanical properties of the material used. Several cadaveric and experimental studies have investigated different stitch patterns to optimize fixation strength and minimize displacement at the meniscus–suture interface [4, 20, 37]. Locking or looped configurations, such as the modified Mason–Allen or double‐locking loop stitches, have been consistently shown to provide greater fixation strength and improved resistance to cyclic loading compared with simple stitches or single‐loop constructs. Although techniques such as the two simple stitches (TSS) may offer reduced displacement under repeated loading, their ultimate failure load is generally lower than that achieved with locking configurations. These findings underline the importance of an adequate suture pattern to maintain hoop tension and minimize gapping at the repair site [44].

Material properties also play a fundamental role in repair stability. Sutures made of UHMWPE demonstrate superior stiffness, higher load‐to‐failure and lower elongation compared with conventional braided sutures [20, 81]. More recently, suture tapes have been introduced as an alternative to cord‐like sutures, showing even greater resistance to displacement and higher tensile strength in biomechanical testing [9, 27, 54, 70, 75]. Nevertheless, their higher cost and limited clinical availability still restrict their widespread use [77].

Overall, combining a strong suture configuration with high‐performance materials appears to be crucial for enhancing the biomechanical stability of MMPRT repairs. However, none of the evaluated constructs have yet fully replicated the mechanical behaviour of the native meniscal root. Detailed biomechanical data from cited studies are summarised in Table S1. A comparative summary of the main advantages and disadvantages of current MMPR repair techniques is provided in Table 4.

**Table 4:** Advantages and disadvantages of MMPR repair techniques.

Technique	Main advantages	Main disadvantages
Pull‐out transtibial repair	Facilitates anatomic reduction and pressure restoration Bone tunnel may enhance biologic healing	Long construct may cause micromotion Risk of suture abrasion and difficulty in proper tensioning
Suture anchor repair	Avoids tibial tunnel, reducing bone trauma Superior cyclic load behaviour and failure strength Less micromotion	May be limited by bone quality Requires good access (posteromedial portal)
All‐inside repair	Minimally invasive and time‐saving Restores near‐physiologic tibiofemoral contact Comparable load to failure to pull‐out	Lower construct stiffness Potential device complications Limited long‐term data
Hybrid techniques (anchor + pull‐out)	Combines direct fixation and retensioning benefits Reduced cyclic displacement (biomechanical studies)	Technically demanding Limited clinical evidence so far
Advanced suture configurations (e.g., Mason–Allen)	Enhanced fatigue resistance and high failure load UHMWPE/tape sutures provide superior strength (up to ~287 N)	Higher technical complexity and cost Limited availability in some centres

Abbreviations: MMPR, medial meniscus posterior root; UHMWPE, ultrahigh‐molecular‐weight polyethylene.

## LIMITATIONS

The present review has some limitations. First, the high heterogeneity of the studies included, along with the low level of evidence, prevents firm conclusions about the reported clinical outcomes. Heterogeneity was assessed narratively, focusing on differences in study design, surgical technique and outcome measures, as quantitative pooling or formal heterogeneity analysis was not feasible given the variability in study types and reported parameters. The primary aim of this systematic review was not to detail the outcomes of these techniques but to provide a broad overview of the surgical methods described in the literature on MMPR repair. Second, there is the potential for article selection bias, which cannot be entirely avoided in advance and may affect the validity and generalizability of this review. The significance of this study lies in its comprehensive review of the surgical techniques documented in the literature for MMPR repair. Surgeons should be aware of this specific type of meniscal lesion and the various surgical techniques available for repair. This knowledge enables the surgeon to select the most appropriate technique for their practice and to follow the evidence related to specific aspects of the surgical process.

## CONCLUSION

MMPR tears can be managed using three main approaches: suture anchor, pull‐out transtibial and all‐inside repair, each with specific advantages and limitations. A modern knee surgeon should be aware of this particular type of meniscal lesion, which, if not appropriately treated, can accelerate degenerative changes. Future research should focus on high‐quality prospective randomized controlled trials to determine the optimal technique that can be considered the gold standard and provide better results.

## AUTHOR CONTRIBUTIONS

*Idea for the article*: Gabriele Cortina and Simone Perelli. *Literature search and data analysis*: Gabriele Cortina and Nicola Pizza. *Manuscript draft*: Gabriele Cortina, Simone Perelli, Nicola Pizza and Vincenzo Condello. *Critical revision of the manuscript*: Vincenzo Madonna and Joan Carles Monllau.

## CONFLICT OF INTEREST STATEMENT

The authors declare no conflicts of interest.

## ETHICS STATEMENT

The authors have nothing to report.

## Supporting information

Table S1.

## Data Availability

Data sharing is not applicable to this article as no data sets were generated or analysed during the current study.
