# Supplementary material for: Medial meniscus posterior root tear repair: An overview of surgical techniques and augmentation strategies
Source: J Exp Orthop. 2026 Jan 11;13(1):e70578. doi: 10.1002/jeo2.70578 (PMC12793040; doi:10.1002/jeo2.70578)
Supplement: Supplementary file 1 — Table S1. [file JEO2-13-e70578-s001.docx]

| Author | Study design | Journal | Purpose | Main outcome |
| --- | --- | --- | --- | --- |
| Kopf S. et al. (2011) | Controlled laboratory study | American Journal of Sport Medicine | Evaluate the maximum failure load of the native meniscal roots and of 3 commonly used meniscal root fixation techniques:   - 2 simple stitches, - modified Kessler stitch - loop stitch | The average maximum failure load of the native meniscal roots was 594 ± 241 N (anterolateral: 692 ± 304 N; posterolateral: 648 ± 140 N; anteromedial: 407 ± 180 N; posteromedial: 678 ± 200 N).  Fixation techniques: the maximum failure load of   - the 2 simple stitches was 64.1 6 ± 22.5 N, - the modified Kessler stitch was 142.6 ±33.3 N, - the loop was 100.9 ± 41.6 N.   None of the fixation techniques recreated the strength of the native roots. |
| Laprade R. et al. (2015) | Controlled laboratory study | American Journal of Sport Medicine | Investigate the cyclic displacement and ultimate failure loads of 4 different meniscus-suture fixation techniques:   - two simple sutures (TSS), - modified Mason-Allen (MMA), - single double-locking loop (S-DLL), - double double-locking loop (D-DLL). | - The TSS group displaced the least (1.78 ± 0.64 mm), followed by the MMA (2.14 ± 0.65 mm), D-DLL (2.97 ± 0.57 mm), and S-DLL (3.81 ± 0.78 mm) groups. - The ultimate failure loads of the MMA (325 ±77 N) and D-DLL (320 ± 50 N) techniques were significantly greater than those of the TSS (192 ± 52 N) and S-DLL (217 ± 51 N) techniques (P < .05). |
| Bachmaier S. et al. (2024) | Controlled laboratory study | The Orthopaedic Journal of Sports Medicine | Evaluate the biomechanical performance of knotless adjustable TPOR in comparison with suture repair techniques with tibial knot tying over a suture button w/different configuration:   - two No. 2 cinch sutures, - two cinch tapes, - two No. 2 simple sutures, - two No. 2 sutures in a Mason-Allen | The 2 cinch sutures fixed technique afforded the lowest (P < .001) initial repair load, stiffness, and relief displacement.  The adjustable TPOR achieved a higher initial repair load (P < .001) and relief displacement (P < .001) than all fixed repairs.  Repairs with adjustable intratunnel fixation showed displacement with cyclic loading similar to the native meniscus. |
| Matthews et al. (2020) | Controlled laboratory study | Knee Surgery & Related Research | Compare the biomechanical properties of nine different suture materials under cyclic and load-to-failure conditions (Ultrabraid®, Ultratape®, Magnum Wire®, TigerWire®, TigerTape®, LabralTape®, Orthocord®, 0 FiberWire®, or 2-0 FiberWire®) | \| **2-0 FiberWire®** \| Elongation: 0.95 ± 0.17 mm (lowest); significantly lower than all others except 0 FiberWire®, TigerWire®, TigerTape®, and LabralTape®. \| \| --- \| --- \| \| **0 FiberWire®** \| Elongation: 1.09 ± 0.17 mm; not significantly different from 2-0 FiberWire®, TigerWire®, TigerTape®, and LabralTape®. \| \| **TigerWire®** \| Elongation: 1.09 ± 0.29 mm; Load to failure: 251.03 ± 25.8 N; Stiffness: 186.49 ± 19.83 N/mm; not significantly different from LabralTape® and TigerTape®. \| \| **TigerTape®** \| Elongation: 1.39 ± 0.29 mm; **Highest load to failure**: 287.43 ± 41.15 N; Stiffness: 173.35 ± 15.60 N/mm; not significantly different from LabralTape® and TigerWire®. \| \| **LabralTape®** \| Elongation: 1.20 ± 0.33 mm; Load to failure: 271.34 ± 48.48 N; **Highest stiffness**: 195.77 ± 49.06 N/mm; not significantly different from TigerWire® and TigerTape®. \| \| **Ultrabraid®** \| Elongation: **1.91 ± 0.34 mm (highest)**; significantly greater than all others except Orthocord® and Magnum Wire®. \| \| **Orthocord®** \| Elongation: 1.59 ± 0.31 mm; intermediate mechanical properties; no significant differences vs Ultrabraid®. \| \| **Magnum Wire®** \| Elongation: 1.43 ± 0.25 mm; intermediate stiffness; no significant differences vs Ultrabraid®. \|   Most sutures failed by **pullout (51%)** or **tearing (45%)**. Tape-based materials (TigerTape®, LabralTape®) exhibited the best combination of strength and stiffness under cyclic loading. |
| Hiranaka T. et al. (2022) | Retrospective comparative study | Knee Surgery & Related Research | Evaluate the clinical outcomes of TPOR of MMPR tears using UHMWPE sutures and suture tape | The arthroscopic meniscal healing scores significantly differed between sutures (mean 6.7 points) and suture tape (mean 7.4 points; p = 0.044). No significant between-group difference in the suture cut-out rate was observed. |
| Stärke C. et al. | Controlled laboratory study | Arthroscopy | measure the tensile forces acting on repaired medial meniscal root lesions and to investigate how they depend on femorotibial rotation, flexion, and compressive load. | Rotation had a highly significant effect on root tension (P < .001). Internal rotation of the femur increased the resultant tension, whereas external rotation decreased it. The tension at the meniscal root was related to the femorotibial load (P < .001). Although no significance was reached, a trend toward higher flexion angles causing more tension was observed. The highest mean tension of 60.1 20.2 N was generated with internal rotation, a 500-N load, and 90 flexion. |

**Table S1**. Summary of biomechanical studies. **MMPR**, medial meniscus posterior root; **TPOR**, Transtibial Pull-out repair, **UHMWPE**, ultrahigh-molecular-weight polyethylene.
